# The multi-biomarker disease activity score tracks response to rituximab treatment in rheumatoid arthritis patients: a post hoc analysis of three cohort studies

**DOI:** 10.1186/s13075-018-1750-5

**Published:** 2018-11-20

**Authors:** Nadia M. T. Roodenrijs, Maria J. H. de Hair, Gill Wheater, Mohsen Elshahaly, Janneke Tekstra, Y. K. Onno Teng, Floris P. J. G. Lafeber, Ching Chang Hwang, Xinyu Liu, Eric H. Sasso, Jacob M. van Laar

**Affiliations:** 1 Department of Rheumatology & Clinical Immunology, University Medical Center Utrecht, Heidelberglaan 100, 3508 GA Utrecht, The Netherlands; 2 Department of Biochemistry, The James Cook University Hospital, Marton Road, Middlesborough, TS4 3BW UK; 3 Department of Rheumatology and Rehabilitation, Suez Canal University, Suez Canal University Circular Road, Ismailia, 411522 Egypt; 4 Department of Nephrology, Leiden University Medical Center, Albinusdreef 2, 2333 ZA Leiden, The Netherlands; 5 Crescendo Bioscience, 341 Oyster Point Blvd, South San Franscisco, CA 94080 USA

**Keywords:** Rheumatoid arthritis, Disease activity, Treatment response, MBDA score, Biomarkers, Rituximab

## Abstract

**Background:**

A multi-biomarker disease activity (MBDA) score has been validated as an objective measure of disease activity in rheumatoid arthritis (RA) and shown to track response to treatment with several disease-modifying anti-rheumatic drugs (DMARDs). The objective of this study was to evaluate the ability of the MBDA score to track response to treatment with rituximab.

**Methods:**

Data were used from 57 RA patients from three cohorts treated with rituximab 1000 mg and methylprednisolone 100 mg at days 1 and 15. The MBDA score was assessed in serum samples obtained at baseline and 6 months. Spearman’s rank correlation coefficients were calculated for baseline values, 6-month values, and change from baseline to 6 months (∆), between MBDA score and the following measures: disease activity score assessing 28 joints (DAS28) using erythrocyte sedimentation rate (ESR) or high-sensitivity C-reactive protein (hsCRP), ESR, (hs)CRP, swollen and tender joint counts assessing 28 joints (SJC28, TJC28), patient visual analogue scale for general health (VAS-GH), health assessment questionnaire (HAQ), and radiographic progression over 12 months using Sharp/van der Heijde score (SHS), as well as six bone turnover markers. Additionally, multivariable linear regression analyses were performed using these measures as dependent variable and the MBDA score as independent variable, with adjustment for relevant confounders. The association between ∆MBDA score and European League Against Rheumatism (EULAR) response at 6 months was assessed with adjustment for relevant confounders.

**Results:**

At baseline, the median MBDA score and DAS28-ESR were 54.0 (IQR 44.3–70.0) and 6.3 (IQR 5.4–7.1), respectively. MBDA scores correlated significantly with DAS28-ESR, DAS28-hsCRP, ESR and (hs)CRP at baseline and 6 months. ∆MBDA score correlated significantly with changes in these measures. ∆MBDA score was associated with EULAR good or moderate response (adjusted OR = 0.89, 95% CI = 0.81–0.98, *p* = 0.02). Neither baseline MBDA score nor ΔMBDA score correlated statistically significantly with ∆SHS (*n* = 11) or change in bone turnover markers (*n* = 23), although ∆SHS ≥ 5 was observed in 5 (56%) of nine patients with high MBDA scores.

**Conclusions:**

We have shown, for the first time, that the MBDA score tracked disease activity in RA patients treated with rituximab and that change in MBDA score reflected the degree of treatment response.

**Electronic supplementary material:**

The online version of this article (10.1186/s13075-018-1750-5) contains supplementary material, which is available to authorized users.

## Background

Rheumatoid arthritis (RA) is the most common, chronic inflammatory joint disease, characterised by synovitis, joint damage, and systemic immune and inflammatory manifestations. Achieving remission or low disease activity is the main treatment goal in order to prevent joint damage and disability [1]. The European League Against Rheumatism (EULAR) and the American College of Rheumatology (ACR) recommend regular assessment of the level of disease activity [2, 3]. The disease activity score assessing 28 joints (DAS28) is one of the most frequently used composite scores for the assessment of disease activity in clinical studies of RA [4]. However, the DAS28 has shortcomings that hamper its use in clinical practice [5]. It does not include the ankles or feet, whereas these are common sites of inflammation in RA. Moreover, the DAS28 contains subjective components, making it highly variable between and within assessors and unreliable at the patient level. In addition, RA inflammation can be extra-articular, which is not readily detected by the DAS28. Thus, there is a need for an objective measure that reflects systemic disease activity and is sensitive to change. It would be of additional benefit if that measure could be used to predict radiographic progression.

The multi-biomarker disease activity (MBDA) score is based on biochemical markers only. It is thus more objective than the DAS28 and may potentially be a better indicator of systemic inflammation. The MBDA score is calculated with an algorithm that uses the concentrations of 12 serum protein biomarkers to produce a score, on a scale of 1 to 100, that represents the level of disease activity in patients with RA [6]. The MBDA score has been validated based on its correlation with DAS28 using C-reactive protein (CRP) and other clinical disease activity measures [7, 8]. The clinical validation of the MBDA score is supported by evidence that the MBDA score is a stronger predictor of radiographic progression than DAS28-CRP, and that it predicts radiographic progression when it is discordant with DAS28-CRP (e.g. when DAS28 is low and MBDA score is high) [9].

The MBDA score tracks response to a variety of disease-modifying anti-rheumatic drugs (DMARDs), including methotrexate [10] (with and without prednisone [11]), tumour necrosis factor (TNF) inhibitors [12–14], abatacept [13–15] and the Janus kinase (JAK) inhibitor tofacitinib [16]. The MBDA score has not yet been assessed in patients treated with rituximab.

Rituximab is an anti-CD20 monoclonal antibody. CD20 is expressed by pre-B and mature B cells, which produce a number of pro-inflammatory cytokines, such as interleukin-6 (IL-6) and TNF. By depleting CD20+ B cells, rituximab treatment leads to a decrease in these pro-inflammatory cytokines [17, 18], thereby reducing clinical disease activity. IL-6 and TNF are 2 of the 12 biomarkers of the MBDA score. It is not known if the clinical response to rituximab is paralleled by changes in the biomarker profile of the MBDA score.

The purpose of the current study was to assess the ability of the MBDA score to measure disease activity upon and track response to treatment with rituximab and, if so, if this would be mainly explained by the objective component of the DAS28 (acute phase reactants). Furthermore, we investigated the ability of the MBDA score to predict radiographic progression and change in serum bone turnover markers upon rituximab treatment.

## Methods

### Study population and treatment protocol

We used data from three prospective cohort studies in which adult, refractory RA patients were treated with rituximab because of active disease despite conventional treatment (e.g. a combination of DMARDs, including maximum tolerable doses of a conventional synthetic (cs)DMARD and/or TNF inhibitor): one cohort from the Leiden University Medical Center (LUMC) [19] and one from the University Medical Center (UMC) Utrecht [20], both in the Netherlands, and the HORUS cohort in the United Kingdom [21]. All patients with available serum samples were selected from the cohorts. Patients received rituximab 1000 mg intravenously on days 1 and 15, after an infusion with intravenous methylprednisolone 100 mg. Patients were followed for at least 1 year from baseline. For the current study, we used disease activity data from the first 6 months following rituximab infusion, to avoid potentially confounding effects from repeat rituximab infusions in some patients.

### Clinical assessments and serum samples

Demographics, disease duration, smoking status (no or yes) and serum status for rheumatoid factor (RF) and for autoantibodies against citrullinated peptides (ACPA) were assessed at baseline. Swollen and tender joint counts assessing 28 joints (SJC28, TJC28), patient visual analogue scale (VAS) for general health (GH), and health assessment questionnaire (HAQ) were obtained for patients at baseline and 6 months, as were erythrocyte sedimentation rate (ESR), CRP and high-sensitivity (hs)CRP (the latter only in HORUS). The DAS28 was calculated using both ESR and hsCRP. EULAR response at 6 months was determined using DAS28-ESR [22]. Radiographs of hands and feet were obtained at baseline and at 12 months (UMC Utrecht cohort) and radiographic progression was assessed using the Sharp/van der Heijde score (SHS) by one reader. Clinically important radiographic progression was defined as ∆SHS ≥ 5 [23]. In the HORUS cohort, serum bone formation markers (BAP (bone-specific alkaline phosphatase), P1NP (procollagen type 1 amino-terminal propeptide), DKK1 (Dickkopf-1), sclerostin) and bone resorption markers (TRAP5b (tartrate-resistant acid phosphatase isoenzyme 5b), βCTX (beta-isomerised carboxy terminal telopeptide of type I collagen)) were determined at baseline and at 6 months (Additional file 1).

### Determination of the MBDA score

Serum samples were collected at baseline in all three cohorts, and at 6 months in the UMC Utrecht and HORUS cohorts. Samples were shipped frozen to Crescendo Bioscience, Inc. (South San Francisco, CA, USA) for measurement of the 12 MBDA biomarkers. The biomarkers represent inflammatory and destructive processes: vascular cell adhesion molecule-1 (VCAM-1), epidermal growth factor (EGF), vascular endothelial growth factor A (VEGF-A), IL-6, TNF receptor type 1 (TNF-R1), matrix metalloproteinase 1 (MMP-1), MMP-3, human cartilage glycoprotein-39 (YKL-40), leptin, resistin, serum amyloid A (SAA) and CRP. The MBDA biomarkers were measured by electrochemiluminescence-based multiplexed sandwich immunoassays (Meso Scale Discovery, Rockville, MD, USA) using the same types of reagents and instrument and the same algorithm as described previously [6, 7].

### Statistical analyses

Baseline characteristics were assessed using descriptive statistics. Differences between the three cohorts were analysed using one-way analysis of variance, Kruskal-Wallis test or chi-square test, as appropriate.

Spearman’s rank correlations (r) were analysed for values at baseline, at 6 months and for change from baseline to 6 months (∆) between MBDA score and the following measures: DAS28-ESR, DAS28-hsCRP, ESR, CRP, hsCRP, SJC28, TJC28, VAS-GH, HAQ, SHS (UMC Utrecht cohort), bone turnover markers (HORUS cohort). Multivariable linear regression analyses were performed using these measures as dependent variable and the MBDA score as independent variable, with adjustment by age, gender, smoking status (no or yes), RF status, ACPA status, and cohort. Bone turnover markers were additionally adjusted for menopausal status (pre-menopausal or post-menopausal) [24]. Logistic regression analysis was performed to assess the association between baseline MBDA score or ∆MBDA score and EULAR response (good or moderate) at 6 months, with adjustment by the same covariates.

Two-sided *p* values < 0.05 were considered statistically significant. All statistical analyses were performed using IBM SPSS Statistics 21 software (IBM Corp, Armonk, NY, USA).

## Results

### Patient characteristics at baseline

Baseline characteristics were generally typical of those for patients with established RA starting rituximab treatment and were mostly similar between the three cohorts. SJC28, ESR, CRP and HAQ were statistically significantly different between the three cohorts (Table 1). Overall, 90% and 80% of patients were seropositive for RF or ACPA, respectively.

**Table 1:** Patient characteristics at baseline

	All, *n = 57*	HORUS, *n = 26*	UMC Utrecht, *n = 20*	LUMC, *n = 11*	*p* value
Female, n (%)	41 (72)	22 (85)	12 (60)	7 (64)	0.15^1^
Age in years, mean (SD)	56.6 (11.2)	59.3 (10.8)	56.7 (11.6)	50.1 (9.5)	0.07^2^
Disease duration in years, median (IQR)	11.5 (6.3–16.4)	9.9 (4.1–14.4)	13.4 (8.4–17.6)	13.0 (5.2–15.5)	0.46^3^
Smoking status, number (%)					
No	37 (65)	16 (62)	12 (60)	9 (82)	0.42^1^
Yes	20 (35)	10 (38)	8 (40)	2 (18)	
RF positive, number (%)	51 (90)	23 (89)	19 (95)	9 (82)	0.51^1^
ACPA positive, number (%)	44 (80)	19 (79), *n = 24*	17 (85)	8 (73)	0.71^1^
Menopausal status, females (%)					
Pre-menopausal	14 (25)	6 (23)	5 (25)	3 (27)	0.30^1^
Post-menopausal	27 (47)	16 (62)	7 (35)	4 (36)	
SJC28, median (IQR)	9 (4–16)	9 (4–15)	12 (8–19)*, n = 19*	4 (1–10)*, n = 8*	0.02^3^
TJC28, median (IQR)	15 (10–23)	16 (11–25)	14 (8–17), *n = 19*	13 (5–24), *n = 8*	0.35^3^
VAS-GH, 0–100 mm (worst), median (IQR)	64 (45–73)	69 (40–78)	57 (46–69), *n = 19*	65 (53–84), *n = 8*	0.36^3^
ESR, mm/h, median (IQR)	37 (21–51)	32 (12–41), *n = 24*	52 (21–91), *n = 18*	32 (29–44), *n = 7*	0.02^3^
CRP, mg/L, median (IQR)	15 (6–34)	11 (5–25), *n = 25*	29 (11–50), *n = 18*	13 (5–56), *n = 5*	0.02^3^
hsCRP, mg/L, median (IQR)	NA	10 (3–26)	NA	NA	NA
DAS28-ESR, median (IQR)	6.3 (5.4–7.1)	6.2 (5.0–7.2), *n = 25*	6.6 (5.8–7.1), *n = 18*	6.1 (3.8–7.3), *n = 8*	0.64^3^
DAS28-hsCRP, median (IQR)	NA	5.8 (4.6–6.8)	NA	NA	NA
MBDA score, median (IQR)	54 (44–70)	51 (44–67), *n = 25*	64 (49–74)	55 (34–71), *n = 7*	0.15^3^
HAQ, median (IQR)	1.8 (1.4–2.1)	1.9 (1.7–2.1)	1.5 (1.1–1.9), *n = 11*	1.3 (1.3–1.9), *n = 7*	0.02^3^
SHS, median (IQR)	44 (24–128)	NA	61 (29–142), *n = 19*	25 (21–94), *n = 8*	0.34^3^

*SD* standard deviation, *IQR* interquartile range, *RF* rheumatoid factor, *ACPA* anti-citrullinated protein antibodies, *SJC28* swollen joint count assessing 28 joints, *TJC28* tender joint count assessing 28 joints, *VAS-GH* patient visual analogue scale for general health, *ESR* erythrocyte sedimentation rate, *mm/h* millimetre/hour, *CRP* C-reactive protein, *mg/L* milligram/litre, *hsCRP* high-sensitivity CRP, *DAS28* disease activity score assessing 28 joints, *MBDA* multi-biomarker disease activity, *HAQ* health assessment questionnaire, *SHS* Sharp/van der Heijde score, *NA* not applicable

^1^Differences between cohorts were analysed using chi-square test

^2^Differences between cohorts were analysed using one-way analysis of variance

^3^Differences between cohorts were analysed using Kruskal-Wallis test

### MBDA score and DAS28 at baseline and 6 months

At baseline the median MBDA score was 54 (interquartile range (IQR) 44–70, *n* = 52), with high (> 44), moderate (30–44) or low (< 30; [7]) scores observed in 40 (77%), 7 (13%) and 5 (10%) patients, respectively. At 6 months the median MBDA score was 51 (IQR 39–58, *n* = 42), with high, moderate or low scores observed in 26 (62%), 11 (26%) and 5 patients (12%), respectively. The median ∆MBDA score was −7 (IQR −19–3, *n* = 42).

At baseline and at 6 months, the median values for DAS28-ESR were 6.3 (IQR 5.4–7.1, *n* = 51) and 5.0 (IQR 4.2–6.2, *n* = 45), respectively, and the median ∆DAS28-ESR was −1.0 (IQR −2.0 to −0.1, *n* = 42). At baseline and at 6 months, the median values for DAS28-hsCRP were 5.8 (IQR 4.6–6.8, *n* = 26) and 4.7 (IQR 3.8–6.2, *n* = 26), respectively, and the median ∆DAS28-hsCRP was − 0.9 (IQR −1.6–0.1, *n* = 26).

### Correlation between MBDA score and disease activity measures

**Fig. 1:**
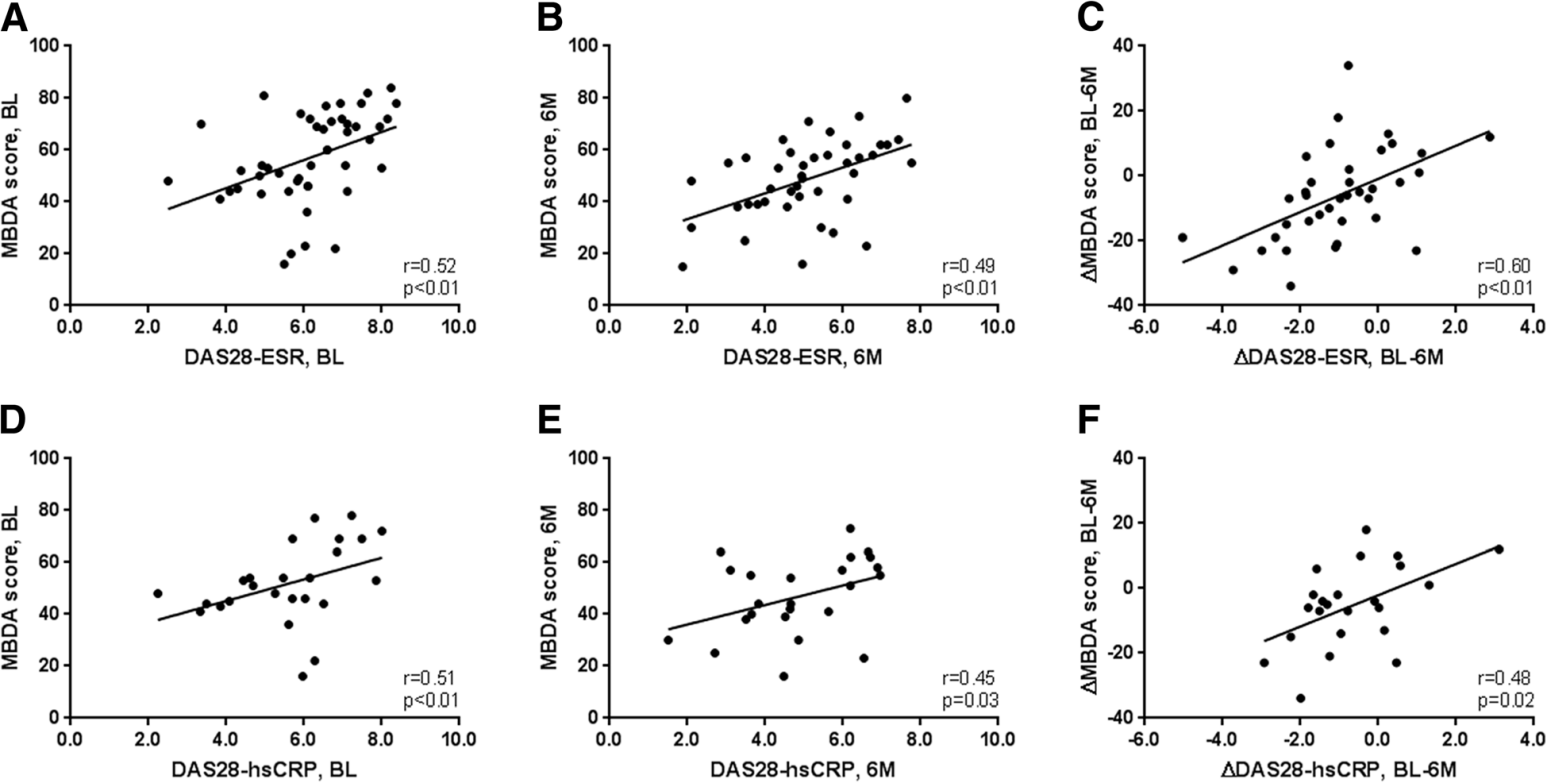
Correlation between MBDA score and DAS28. **a** MBDA score versus DAS28-ESR at baseline (*n* = 46). **b** MBDA score versus DAS28-ESR at 6 months (*n* = 42). **c** ∆MBDA score versus ∆DAS28-ESR, from baseline to 6 months (*n* = 38). **d** MBDA score versus DAS28-hsCRP at baseline (*n* = 25). **e** MBDA score versus DAS28-hsCRP at 6 months (*n* = 24). **f** ∆MBDA score versus ∆DAS28-hsCRP, from baseline to 6 months (*n* = 23). Negative change values represent improvement over 6 months

Correlations between MBDA score and DAS28 and their changes over time are shown in Fig. 1. A significant Spearman’s correlation was found between MBDA score and DAS28-ESR at baseline (*r* = 0.52, *p* < 0.01) and at 6 months (*r* = 0.49, *p* < 0.01). ∆MBDA score from baseline to 6 months was significantly correlated with ∆DAS28-ESR (*r* = 0.60, *p* < 0.01).

Similarly, the MBDA score was significantly correlated with DAS28-hsCRP at baseline (*r* = 0.51, *p* < 0.01) and at 6 months (*r* = 0.45, *p* = 0.03). ∆MBDA score from baseline to 6 months was significantly correlated with ∆DAS28-hsCRP (*r* = 0.48, *p* = 0.02).

MBDA score was significantly correlated with ESR, hsCRP and CRP, as was also true for their changes from baseline to 6 months (Table 2).

**Table 2:** Correlations and associations between the MBDA score and disease activity measures

Measure	Time point or period for comparison with MBDA score	Number of available samples	r	*p* value	β (95% CI)^1^	*p* value
DAS28-ESR	BL	46	0.52	< 0.01	0.05 (0.02–0.07)	< 0.01
	6 M	42	0.49	< 0.01	0.06 (0.02–0.09)	0.01
	∆	38	0.60	< 0.01	0.05 (0.01–0.08)	0.02
ESR	BL	44	0.75	< 0.01	1.20 (0.71–1.70)	< 0.01
	6 M	42	0.66	< 0.01	0.81 (0.36–1.26)	< 0.01
	∆	37	0.48	< 0.01	0.57 (−0.03–1.17)	0.06
DAS28-hsCRP^2^	BL	25	0.51	< 0.01	0.06 (0.02–0.10)	0.01
	6 M	24	0.45	0.03	0.06 (0.02–0.10)	< 0.01
	∆	23	0.48	0.02	0.05 (0.00–0.09)	< 0.05
hsCRP^2^	BL	25	0.80	< 0.01	1.24 (0.72–1.76)	< 0.01
	6 M	24	0.80	< 0.01	0.75 (0.41–1.10)	< 0.01
	∆	23	0.71	< 0.01	0.90 (0.60–1.21)	< 0.01
CRP	BL	46	0.75	< 0.01	1.07 (0.62–1.52)	< 0.01
	6 M	40	0.76	< 0.01	0.82 (0.58–1.06)	< 0.01
	∆	37	0.59	< 0.01	0.68 (0.18–1.19)	< 0.01
SJC28	BL	48	0.15	0.32	0.10 (−0.06–0.26)	0.22
	6 M	42	0.26	0.10	0.14 (−0.01–0.28)	0.06
	∆	40	0.42	< 0.01	0.12 (−0.04–0.29)	0.14
TJC28	BL	48	0.23	0.12	0.17 (0.02–0.32)	0.03
	6 M	42	0.25	0.11	0.17 (−0.01–0.34)	0.06
	∆	40	0.28	0.08	0.04 (−0.15–0.23)	0.67
VAS-GH	BL	48	0.20	0.18	0.34 (−0.12–0.79)	0.14
	6 M	42	0.27	0.09	0.46 (−0.08–0.99)	0.09
	∆	40	0.36	0.02	0.74 (0.08–1.40)	0.03
HAQ	BL	39	0.02	0.91	0.06 (−0.06–0.02)	0.30
	6 M	41	−0.03	0.85	−0.01 (− 0.01–0.01)	0.84
	∆	34	0.19	0.28	0.00 (−0.01–0.01)	0.77

*DAS28* disease activity score using 28 joints, *ESR* erythrocyte sedimentation rate, *hsCRP* high-sensitivity C-reactive protein, *SJC28* swollen joint count assessing 28 joints, *TJC28* tender joint count assessing 28 joints, *VAS-GH* patient visual analogue scale for general health, *HAQ* health assessment questionnaire, *MBDA* multi-biomarker disease activity, *BL* MBDA score and measure both at baseline, *6 M* MBDA score and measure both at month 6, ∆ change in MBDA score and measure, both from baseline to month 6, *r* Spearman’s rank correlation, *CI* confidence interval

^1^β: regression coefficient from multivariable linear regression analysis, after adjustment by age, gender, smoking status, RF status, ACPA status, and cohort

^2^HORUS cohort only

Correlations were not significant between the MBDA score and SJC28, TJC28, VAS-GH or HAQ, except for ∆SJC28 and ∆VAS-GH from baseline to 6 months (Table 2).

The results of the multivariable regression analysis resembled those of the correlation analyses, except that the associations between ∆MBDA score versus ∆ESR and ∆SJC28 were not statistically significant and the association between MBDA score versus TJC28 at baseline was statistically significant (Table 2).

### Association between MBDA score and EULAR response

**Fig. 2:**
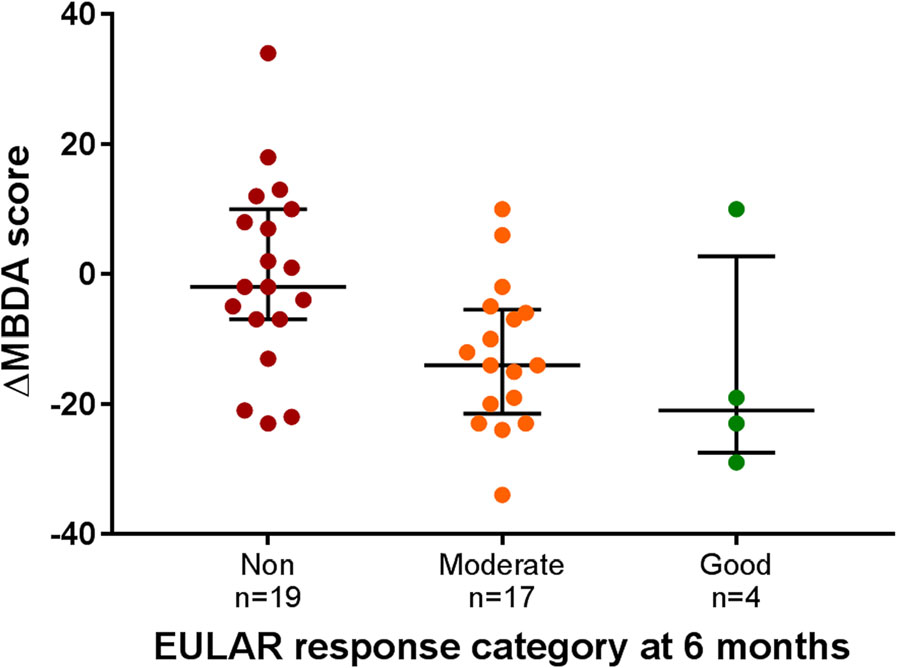
Change in MBDA score by EULAR response category at 6 months. Individual patient values of ∆MBDA score from baseline to 6 months are shown as *dots*, grouped by EULAR response category at 6 months (EULAR non-, moderate, good response). *Dark lines* represent median values. *Whiskers* represent interquartile ranges (25th–75th). ∆: change

At 6 months, 21 patients (48%) were classified as non-, 19 patients (43%) as moderate and 4 patients (9%) as good EULAR responders. The distribution of values for ∆MBDA score within each EULAR response category is shown in Fig. 2. ∆MBDA score from baseline to 6 months was significantly associated with EULAR response (good or moderate) versus non-response at 6 months (odds ratio (OR): 0.93 (95% CI = 0.88–0.98, *p* = 0.01) per unit change in MBDA score, Fig. 2). Adjusted by age, gender, smoking status, RF status, ACPA status, and cohort, this association remained statistically significant (OR: 0.89 (95% CI = 0.81–0.98, *p* = 0.02) per unit change in MBDA score).

The MBDA score at baseline was not associated with EULAR response (good or moderate) versus non-response at 6 months, with OR of 1.01 (95% CI = 0.98–1.05, *p* = 0.35) per unit MBDA score, even after adjustment by age, gender, smoking status, RF status, ACPA status, and cohort (OR: 1.03 (95% CI = 0.98–1.08, *p* = 0.27) per unit MBDA score).

### Correlation between MBDA score and radiographic progression or bone turnover markers

For the 11 patients with radiographs available at baseline and 12 months, all from the UMC Utrecht cohort, the median ∆SHS was 3 (IQR −1–12). At baseline, low, moderate and high MBDA scores were observed in 1, 1 and 9 patients, respectively. Radiographic progression (∆SHS ≥ 5) in patients with low, moderate and high MBDA scores was observed in 0 (0%), 0 (0%) and 5 (56%) patients, respectively.

No significant Spearman’s correlation was found between MBDA score or ∆MBDA score and ∆SHS over 12 months, nor bone turnover markers (Table 3). Similar findings were obtained with multivariable regression analysis adjusted by age, gender (menopausal status for bone turnover markers), smoking status, RF status and ACPA status (Table 3).

**Table 3:** Correlations and associations between the MBDA score and radiographic progression or bone turnover markers

Measure	Time point or period for comparison with MBDA score	Number of available samples	r	*p* value	β (95% CI)^1^	*p* value
∆SHS (baseline-12 months)^2^	BL	11	0.19	0.57	0.34 (−0.33–1.01)	0.23
	6 M	11	0.18	0.60	0.18 (−0.72–1.08)	0.62
	∆	11	0.19	0.57	−0.38 (−1.35–0.60)	0.34
Bone turnover markers^3^						
βCTX	∆	23	0.22	0.31	1.10 (−6.79–8.99)	0.77
P1NP	∆	23	−0.14	0.54	−0.31 (−2.05–1.42)	0.70
BAP	∆	23	−0.01	0.98	−0.01 (− 0.17–0.15)	0.88
TRAP5b	∆	23	−0.20	0.37	0.00 (−0.03–0.03)	0.99
DKK1	∆	23	0.27	0.22	0.15 (−0.40–0.69)	0.57
Sclerostin	∆	23	0.11	0.63	0.10 (−0.37–0.56)	0.66

*SHS* Sharp/van der Heijde score, *βCTX* beta-isomerised carboxy terminal telopeptide of type I collagen, *P1NP* procollagen type 1 amino-terminal propeptide, *TRAP5b* tartrate-resistant acid phosphatase isoenzyme 5b, *DKK1* Dickkopf-1, *BAP* bone-specific alkaline phosphatase, *MBDA* multi-biomarker disease activity, *BL* MBDA score at baseline, *6 M* MBDA score at month 6, ∆ change (for SHS: ∆MBDA score from baseline to month 6 and ∆SHS from baseline to month 12; for bone turnover markers: both ∆ from baseline to month 6), *r* Spearman’s rank correlation, *CI* confidence interval

^1^β: regression coefficient from multivariable linear regression analysis, after adjustment by age, gender and/or menopausal status, smoking status, RF status, and ACPA status

^2^UMC Utrecht cohort only

^3^HORUS cohort only

## Discussion

The MBDA score has been shown to track response to a variety of DMARDs. We found significant correlations between the MBDA score and DAS28-ESR as well as DAS28-hsCRP at baseline and at 6 months, and between ΔMBDA score and ΔDAS28-ESR and ΔDAS28-hsCRP from baseline to 6 months in patients treated with rituximab. Moreover, ΔMBDA score was significantly associated with EULAR response to rituximab treatment. This is the first time it has been shown that the MBDA score can be used to track disease activity in RA patients upon treatment with rituximab and that change in the MBDA score reflects response to rituximab treatment.

Our findings on the MBDA score are consistent with several previous studies in RA patients upon treatment with other cs-, biological, or targeted synthetic DMARDs [6–8, 10–16].

In our study, we additionally investigated if the MBDA score correlated with the individual components of the DAS28. We found correlations between the MBDA score or ΔMBDA score and ESR, hsCRP or their changes, but found limited correlations between the MBDA score and the other DAS28 components. The correlation between the MBDA score and the DAS28 thus seems predominantly dependent on the biochemical components of the DAS28, the ESR or (hs)CRP. It would be of interest to assess the additional value of the MBDA score above ESR or CRP alone, but the present study was not powered to analyse this. A larger study has reported that an increase in TJC, SJC and patient global assessment was paralleled by an increase in MBDA score; and that, in patients positive for either RF and/or ACPA, an MBDA score excluding CRP was a significant predictor of both DAS28-CRP, and of DAS28 without any CRP or ESR component [7].

In addition, MBDA score appeared to be more sensitive for detecting inflammation than ESR or CRP. A study of 9135 RA patients with active disease found that ESR and CRP were normal in the majority [25]. In other studies, MBDA score was often elevated in such patients [26] and, when it was, risk of radiographic progression was increased [9, 27]. In patients with disproportionally high subjective disease activity components (e.g. high tender joint counts with low ESR or CRP) the MBDA score might be an important alternative disease activity measure. We could not address this hypothesis, as no patients with normal ESR or normal CRP (defined as ≤ 1 mm/h or mg/L) were included in this study.

Previous studies have shown that the MBDA score was a significant predictor of radiographic progression, both in early and established RA [9, 27–30]. In the present study, all patients with clinically important radiographic progression (ΔSHS ≥ 5) had a high MBDA score at baseline. This result resembles the findings in previous studies [9, 27–30]. We did not find a significant Spearman’s correlation between baseline MBDA score and ΔSHS in patients treated with rituximab, possibly due to the small number of patients (*n* = 11) and the limited observation period.

B-cell depletion upon rituximab treatment has been shown to be most effective in RF-positive patients [31], and has been suggested to be associated with ACPA positivity [32]. In future studies, it would be of interest to stratify the performance of the MBDA score in rituximab-treated RA patients according to RF and ACPA status.

Other studies have shown that rituximab treatment increases bone formation and decreases bone resorption in RA patients [33, 34]. For example, a significant correlation was found between the changes of DAS28 and βCTX [34], showing that the anti-inflammatory therapeutic response with rituximab and the anti-resorptive effect on bone might be related. In future, in larger studies with longer follow-up, it may be of interest to investigate the relationship between the MBDA score and bone turnover.

## Conclusions

In conclusion, we have shown, for the first time, that the MBDA score correlated with DAS28 following treatment with the B-cell depleting agent rituximab and that ΔMBDA score reflected the treatment response. Our findings are consistent with previous research in RA patients treated with other DMARDs.

## Additional file

Additional file 1: Determination of bone turnover markers. (PDF 102 kb)

## Abbreviations

ACPA: Autoantibodies against citrullinated peptides
ACR: American College of Rheumatology
BAP: Bone-specific alkaline phosphatase
CI: Confidence interval
CRP: C-reactive protein
DAS28: Disease activity score assessing 28 joints
DKK1: Dickkopf-1
DMARDs: Disease modifying anti-rheumatic drugs
EGF: Epidermal growth factor
ESR: Erythrocyte sedimentation rate
EULAR: European League Against Rheumatism
HAQ: Health assessment questionnaire
hsCRP: High-sensitivity C-reactive protein
IL-6: Interleukin 6
JAK: Janus kinase
MBDA: Multi-biomarker disease activity
MMP-1: Matrix metalloproteinase 1
MMP-3: Matrix metalloproteinase 3
OR: Odds ratio
P1NP: Procollagen type 1 amino-terminal propeptide
RA: Rheumatoid arthritis
RF: Rheumatoid factor
SAA: Serum amyloid A
SD: Standard deviation
SHS: Sharp/van der Heijde score
SJC28: Swollen joint count assessing 28 joints
TJC28: Tender joint count assessing 28 joints
TNF: Tumour necrosis factor
TNF-R1: TNF receptor type 1
TRAP5b: Tartrate-resistant acid phosphatase isoenzyme 5b
VAS-GH: Visual analogue scale measuring general health
VCAM-1: Vascular cell adhesion molecule-1
VEGF-A: Vascular endothelial growth factor A
YKL-40: Human cartilage glycoprotein-39
βCTX: Beta-isomerised carboxy terminal telopeptide of type I collagen
