# Supplementary material for: The multi-biomarker disease activity score tracks response to rituximab treatment in rheumatoid arthritis patients: a post hoc analysis of three cohort studies
Source: Arthritis Res Ther. 2018 Nov 20;20:256. doi: 10.1186/s13075-018-1750-5 (PMC6245625; doi:10.1186/s13075-018-1750-5)
Supplement: Supplementary file 1 — Determination of bone turnover markers. (PDF 102 kb) [file 13075_2018_1750_MOESM1_ESM.pdf]

Additional file 1

Determination of bone turnover markers

All bone turnover markers were quantified following the manufacturer's guidelines and reference ranges (BAP: 1-75µg/L; P1NP: premenopausal females 15.1–58.6 µg/L, postmenopausal females 16.3–73.9 µg/L, males >50 years 15.1– 58.6 µg/L; DKK1: 1.7-160pmol/L; sclerostin: 7.5-240pmol/L; TRAP5b: 0.5-10.0U/L; βCTX: premenopausal females 25–573 ng/L, postmenopausal females 104–1008 ng/L, males >50 years 0–854 ng/L). BAP was quantified in 50µl serum by chemiluminescence on the IDS iSYS analyser (ImmunoDiagnostic Systems - Boldon, Tyne and Wear, UK). P1NP and βCTX were quantified by electrochemiluminescent immunoassay on the Elecsys 2010 analyser (Roche Diagnostics, Lewes, UK). DKK-1 and sclerostin levels were measured in 20µl serum using a manual Biomedica ELISA, purchased from Oxford Biosystems Cadama (Wheatley, Oxford, UK), a UK distributor for Biomedica Medizinprodukte (Vienna, Austria). TRAP-5b was measured in 100µl serum by a manual IDS ELISA (ImmunoDiagnostic Systems - Boldon, Tyne and Wear, UK). This method specifically measured TRAP isoform 5b activity freshly liberated from osteoclasts.
